# Seaweed Polysaccharide in Food Contact Materials (Active Packaging, Intelligent Packaging, Edible Films, and Coatings)

**DOI:** 10.3390/foods10092088

**Published:** 2021-09-03

**Authors:** Kalpani Y. Perera, Shubham Sharma, Dileswar Pradhan, Amit K. Jaiswal, Swarna Jaiswal

**Affiliations:** 1 School of Food Science and Environmental Health, College of Sciences and Health, Technological University Dublin—City Campus, Central Quad, Grangegorman, Dublin D07 ADY7, Ireland; kalpani.gamage@TUDublin.ie (K.Y.P.); shubham.sharma@TUDublin.ie (S.S.); d20127371@mytudublin.ie (D.P.); swarna.jaiswal@TUDublin.ie (S.J.); 2 Environmental Sustainability and Health Institute (ESHI), Technological University Dublin—City Campus, Grangegorman, Dublin D07 H6K8, Ireland

**Keywords:** seaweeds, polysaccharide, active packaging, intelligent packaging, edible films, coating, legislations

## Abstract

Food contact materials (FCMs) are materials that come in contact with food products such as food packaging which play a significant role in the food quality and safety. Plastic, which is a major food packaging material, harms the eco-system, wildlife, and the environment. As a result, numerous researches have been in progress on alternative polymers, which has similar properties as plastic but is also environmentally friendly (biodegradable). In recent years, the utilization of seaweed polysaccharides has piqued interest due to its biodegradability, non-toxicity, antioxidant capabilities, and excellent film formation ability. However, it has a number of drawbacks such as low tensile strength, water solubility, and moderate antibacterial characteristics, among others. The addition of other biopolymers, nanoparticles, or natural active agents improves these features. In this review article, we have summarized the current state of seaweed polysaccharide research in active packaging, intelligent packaging, edible films, and coatings. It also highlights the physical, thermal, antioxidant, and other properties of these materials. Finally, the article discusses the relevant legislation as well as the field’s future prospects. Research shows that seaweeds polysaccharide looks promising as a sustainable food contact material, but there is always a potential for development to make it market feasible.

## 1. Introduction

Food contact materials (FCMs) are materials that come in contact with food products, including food packaging, containers, cutlery, and dishes. These materials play a significant role in the food industry starting from production to storage to distribution. It allows food products to maintain their standard quality and ensure the food products’ safety while following the necessary government regulations and policies. The safety of the manufactured FCMs is being assessed because the chemicals in these materials can migrate into food products. The FCMs currently available on the market are mostly plastic, rubber, paper, and metal [1]. In 2019, Europe produced 50.7 million tons of plastic, 39.6% of which was used in the food packaging industry. Out of the total plastic produced, a total of 29.1 million tons of plastic waste per year (in 2018), out of which 24.9% is found in landfills [2]. This leads to the rapid accumulation of plastic waste in landfills and gradual accumulation in the ocean. Plastic waste harms the eco-system, wildlife, and the environment. Further, microplastics and the toxic additives of plastic flow through the food chain. Due to the drawbacks of current FCMs, the use of biodegradable polymers is an option. Additionally, because of their numerous beneficial properties, seaweeds have gained popularity as a -FCMs due to the increasing demand for biopolymers.

Seaweeds are a multicellular, macroscopic, benthic algae with a fast growth rate, resulting in a rapid accumulation of biomass. They are available in abundance, which is the main reason for their high demand in the food industry. It is not only utilized in the food industry but also in the agriculture, pharmaceutical, and biofuel industries. The seaweeds can be mainly categorized into three groups: red seaweeds (e.g., *Poryphyra capensis*, *Aeodes orbitosa*, *Notogenia stiriata-* polysaccharide—agar, carrageenan), brown seaweeds (e.g., *Laminaria pallida*, *Fucus* and *Zonaria* species—polysaccharide—alginate, fucoidan, laminarin), and green seaweeds (e.g., *Cladophora*, *Ulva* and *Monostroma* species-Polysaccharide-Ulvan) [3]. These seaweeds have numerous bioactive compounds, such as polysaccharides (e.g., alginate, laminarans, and fucoidans), protein (e.g., phycobiliproteins), minerals, vitamins, unsaturated essential fatty acids, polyphenols (e.g., phlorotannins and bromophenols), carotenoids (e.g., fucoxanthin, and astaxanthin), tocopherol, antioxidants, and antimicrobial agents, which are an additional quality as FCMs [4,5]. The properties of the seaweeds depend upon their different extraction procedures [6].

**Figure 1:**
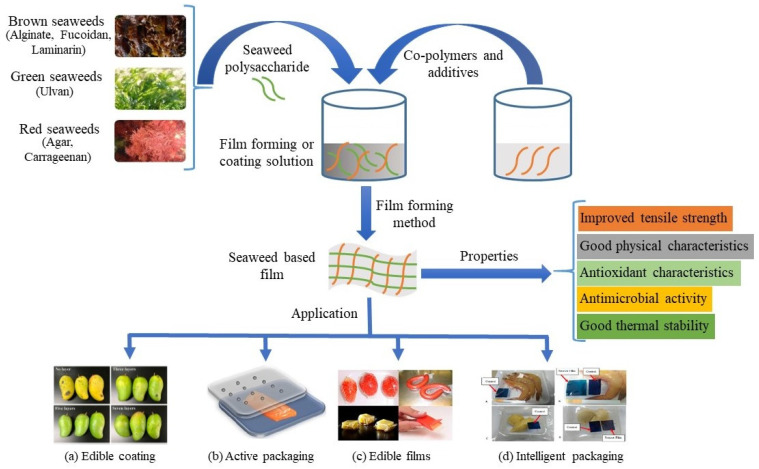
The mechanism for developing seaweed-based food contact materials. (**a**) Mangoes coated in chitosan- and alginate-based coatings enriched with cinnamon essential oil microcapsules [13], (**b**) Active packaging of cold-smoked salmon with Zn-MgO/Alginate film [14], (**c**) Some examples of edible films [15], (**d**) Intelligent packaging of shrimp and durian using ι-carrageenan based colorimetric pH sensor film [16].

Seaweeds can be utilized as FCMs as a potentially active agent, polysaccharide rich raw material, or extracts [5]. Seaweed-based products can improve the materials’ sustainability, sensory properties, and functionality. Seaweeds are used in active packaging, intelligent packaging, edible films, edible coatings, sachets, and in other utensils. To be used as useful FCMs, seaweeds are combined with other polysaccharides, nanoparticles, essential oils, or plant extracts to improve their barrier, thermal, mechanical, antioxidant, and antimicrobial properties [6]. Seaweeds have been incorporated with other polymers or with seaweed polymers to form active packages such as alginate- sepiolite—myrtle berries extract [7], sodium alginate- β-cyclodextrin- carvacrol [8], agar- sodium alginate- SiO_2_ nanoparticle [9], furcellaran- gelatin hydrolysate-rosemary extract [10], etc (explained in detail at later section). With the incorporation of mulberry [11], anthocyanin [12], and the ability to change color at different pH sensitivities, seaweeds have also been used as intelligent packaging systems. Seaweed’s non-lethal, non-toxic properties, as well as its film-forming abilities, have led to its use as edible films and coatings. The mechanism for developing these seaweed-based FCMs has been demonstrated in Figure 1.

The nature and the beneficial properties of seaweeds have led them to be used as industrial level FCMs presently. Marine Innovation, Ulsan, Korea, has developed eco-friendly egg trays, cartons, fruit trays, paper cups, coffee carriers, and disposable paper plates from seaweed/seaweed products. Kelpn Limited, Christchurch, New Zealand, is currently using Kelp seaweed based alternative packaging materials that are fully biodegradable. Evoware, Indonesia, has developed biodegradable edible films from seaweed. These edible sachets are used for the packaging of various food products such as cereal, coffee, salt, pepper, sauces, and oils. While the edible wraps are used to wrap burgers, sandwiches, and rice. Notpla, London, UK, developed Ooho, which is used as an edible water bottle from seaweed for the packaging of water. Further, they also developed Lucozade seaweed edible capsules. JetNet Corporation, Sewickley, PA, USA, developed an edible Nutrafilm from Carrageenan that increases the shelf life of meat, poultry, and dairy products. These packaging materials are edible or biodegradable in 4–6 weeks [17].

In this review article, we have aimed at highlighting current state of research into seaweed-based food contact materials. Further, it summarizes the different effects such as mechanical, physical, chemical, thermal, antimicrobial, and antioxidant properties of seaweed based FCMs. Furthermore, it emphasizes the legal aspects of seaweeds as FCMs, as well as future prospects.

## 2. Seaweeds

Seaweeds are usually found on rocks, pebbles, shells, and seawater plants in shallow waters at a depth of 180 m. It has a rapid growth rate and is highly abundant. Seaweeds are mainly used as food products, fertilizers, animal feed, and medicine. Seaweed consumption has increased by about 176% since 1995. Due to its multifunctional applications, high cost ~USD 50–80/ton, and the trend of being used as sustainability products, seaweed cultivation is currently in demand. However, seaweed cultivation and farming are very limited as they are presently confined to coastal areas [18].

As mentioned above, the seaweeds are classified into green seaweeds (Chlorophyceae), red seaweeds (Rhodophyceae), and brown seaweeds (Phaeophyceae) depending upon the pigmentation. The green seaweed species *Cladophora*, *Ulva*, and *Monostroma* produce the acidic polysaccharide Ulvan. Red seaweeds *Poryphyra capensis*, *Aeodes orbitosa*, *Notogenia stiriata* widely produce Agar and Carrageenan. While brown seaweeds *Laminaria pallida*, *Fucus*, and *Zonaria* species produce the polysaccharides Alginate, Fucoidan, and Laminarin. All species of seaweeds have high carbohydrate (accounting for about 40% of the mass) content, while brown seaweed also has a high protein content [18].

The most commonly used seaweed polysaccharides as FCMs are Alginate, Agar, and Carrageenan, as shown in Table 1. Alginate consists of D-mannuronic acid and L-guluronic acid (G) in varied molecular weights, proportions, and configurations. The properties of alginate depend upon the ratio of mannuronic acid and guluronic acid. When alginate consists of a higher amount of guluronic acid, there is a high ability to form strong bonds. In contrast, if the guluronic acid levels are low, a softer and more flexible structure is formed. As a result of the chemical structure alginate exhibits unique colloidal properties, this aids in stabilizing and thickening of films or coating. Alginate is used as a coating material because of its propensity to react irreversibly and efficiently with divalent and trivalent metal cations to create water-insoluble polymers. Alginate has good film-forming ability with high transparency and uniformity. Further, it is impermeable to fats and oils. Carrageenan is a hydrophilic polymer consisting of a linear chain of partially sulphated galactans. It is mainly made up of three types: kappa carrageenan, iota carrageenan, and lambda carrageenan. With potassium salts, kappa carrageenan produces strong gels; with calcium salts, it produces brittle gels. Kappa carrageenan coatings efficiently protect vegetables and fruit from moisture loss and oxidation. Agar is formed from a mixture of agarose and agaropectin. Agar is primarily composed of L and D galactose units that are alternated regularly. Agarose is responsible for the gelling properties of agar, making it suitable to form films and coatings. Agar can generate transparent, robust, thermoreversible gels which are water in soluble [19]. The biocompatibility, gel-forming ability, emulsification, gelation, and foaming) capabilities of various seaweed polysaccharides are based on their unique structure. In addition to the properties of the seaweed, its nutritional value including minerals, vitamins, calories, and antioxidants, are beneficial in making edible films and coatings.

## 3. Seaweeds Application as Food Contact Materials

### 3.1. Active Packaging

Active Packaging is an innovative approach that can be characterized as a form of packaging in which the package, the product, and the environment interact to extend shelf life, improve safety, and enhance sensory characteristics while sustaining product quality. Active packaging is defined as packaging systems that interact with food in such a manner that they “deliberately contain components that would release or absorb substances into or from the packed food or the environment surrounding the food”, according to European regulation (EC) No 450/2009 [20]. The active packaging method combines the preservation properties of antimicrobials and other ingredients with pre-existing food packaging concepts [21]. Food-derived chemicals are released into the food or the environment by the packaging surrounding the food, or food-derived chemicals are absorbed by the packaging surrounding the food. There are two types of active packaging systems: active scavenging systems (absorbers) and active-releasing systems (emitters). While the absorber removes undesirable molecules from food or its surroundings, such as moisture, carbon dioxide, oxygen, ethylene, or odor, the emitters add compounds to packed food or the headspace, such as antimicrobial compounds, flavors, ethylene, carbon dioxide, antioxidants, or ethanol [22].

The use of active packaging instead of direct addition to the food to add active ingredients such as antimicrobials and antioxidants may reduce the amount of these substances needed. Meal deterioration or microbial development occurs on the surface of most fresh and processed foods. Hence, active chemicals are traditionally introduced to the bulk of the food. Furthermore, when active chemicals are directly added to food, their activity may be inhibited or reduced as a result of interactions between the active substances and the food components, and/or during food processing. As a result, adding active ingredients via active packaging may be more effective than adding them to the bulk of the food. Many active food packaging systems are now being developed, including moisture control packaging, oxygen scavengers, carbon dioxide generating systems, antimicrobial active packaging, ethylene scavengers, and flavor and odor absorbent packaging [23,24].

Oxygen scavengers (OS) are the most common type of active packaging, and they work by removing oxygen from the environment and preventing it from reacting with the product. Vacuum packaging is one of the methods of preserving food in an oxygen-free atmosphere [25]. Modified atmosphere packaging (MAP), a technique for altering the environment of packaging, is another method for removing oxygen from the headspace of food packaging. In comparison to vacuum packing, which focuses primarily on removing oxygen from the headspace, MAP is a more versatile approach [26]. Other oxygen scavenging packaging methods are iron-based scavenging systems, platinum group metals-based scavenging systems, unsaturated hydrocarbon-based scavenging systems, α-tocopherol based scavenging systems, ascorbic acid-based scavenging systems, enzyme-based scavenging systems, and micro-organisms-based scavenging systems. However, it has been found that vacuum packaging or MAP can reduce the residual oxygen content in the headspace to only 0.5–2 vol%, which could be harmful. Oxygen scavengers, on the other hand, may lower oxygen levels to less than 0.1 vol% percent, resulting in a longer shelf life [26,27].

By integrating or coating antimicrobial chemicals in/on food packaging, antimicrobial packaging tends to decrease, inhibit, or delay the growth of microorganisms, hence extending the lag phase and reducing microorganism growth. One of the approaches is by incorporating active compounds within the package via a pad, tablet, or sachet, and allowing mechanisms such as evaporation and absorption to limit microbial growth and other degrading processes, the interior environment of the packaging can be altered. Another approach is to directly incorporate an antimicrobial agent into the polymer and then slowly released it into the packing headspace or onto the food surface. Additionally, a third option is to coat the packaging with a matrix that acts as a carrier for the antimicrobial chemical, which would then either evaporate into the atmosphere or diffuse into the food.

**Figure 2:**
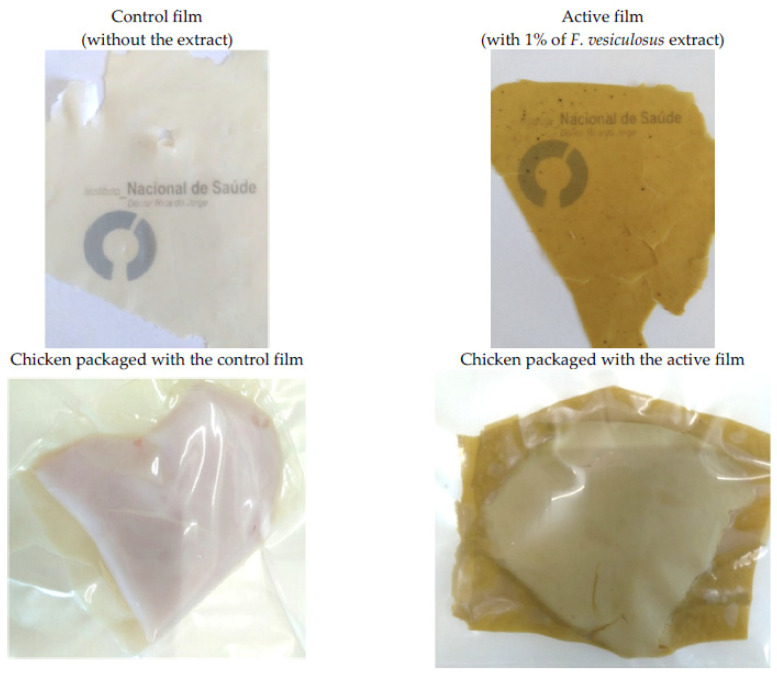
Whey protein control film and active film with 1% *Fucus vesiculosus* (brown seaweed) extract [29].

Antimicrobial polymers include Ferulic acid incorporated poly l-lactic acid/polybutylene adipate terephthalate, which has shown promising results as a novel active food packaging film due to its antibacterial properties against *L. monocytogenes* and *E. coli* [28]. Enzymes (e.g., lysozyme), essential oils (e.g., clove essential oil, orange oil), organic acids (e.g., lactic acid, acetic acid), and nanoparticles are examples of antimicrobial agents (e.g., zinc, silver, titanium). Andrade et al. [29] have formed a whey protein concentrate active coating with *Fucus vesiculosus* extract to inhibit chicken breasts from oxidizing their lipids as demonstrated in Figure 2. They observed an increase in thickness, tensile strength, and elastic modulus. The active film also prevented lipid oxidation in chicken breasts for 25 days of storage. Han and Wang [30] have prepared an antimicrobial film with sodium alginate and carboxymethyl cellulose, glycerin, CaCl_2_, and the natural antibacterial ingredient pyrogallic acid (PA). Their observations suggest an increase in the UV barrier property, moisture content, water vapor permeability, and oxygen permeability. Additionally, the films with a high concentration of PA were more efficient against *E. coli* and *S. aureus*. Further studies on seaweed-based food contact materials are depicted in Table 2.

### 3.2. Smart and Intelligent Packaging

Intelligent packaging is a type of packaging that keeps track of a product’s quality, safety, and location during transportation, storage, retail sales, and use. Intelligent packaging materials are defined by the European Food Safety Authority (EFSA) as “materials and items that monitor the state of packaged food or the environment surrounding the food” [34]. They could transmit the packed product’s conditions, but they do not interact with it [23]. Experts predict that the next generation of intelligent packaging will be the future of food packaging. As intelligent packaging technologies transform traditional packaging communication functions into intelligent communication, they have contributed to a further major shift in the existing perception of packaging.

Two systems are the most common types of intelligent packaging [35]. The first is based on measuring factors outside of the packaging, whereas the second directly assesses the quality of the product inside packaging and may come into direct contact with food, necessitating additional food safety and quality controls [36]. In general, data carriers, indicators, and sensors are the three basic technologies employed in intelligent packaging systems (as portrayed in Table 3) [37].

#### 3.2.1. Data Carriers

Data carriers aid in the effective movement of information throughout the supply chain. The purpose of data carriers is to ensure traceability, automation, theft protection, and imitation protection [38]. Data carriers store and send information on storage, distribution, and other factors in order to ensure this. As a result, they are frequently found on tertiary packaging. Barcode labels and RFID (Radio Frequency Identification) tags are the most commonly used data carriers [37].

#### 3.2.2. Indicators

Indicators detect the existence or absence of a material, the extent of an interaction between several substances, or the concentration of a specific substance. Direct changes, such as varying color intensities, are used to visualize this information [39]. They are positioned inside or outside of the package based on the indicator [37,38]. The most prevalent types of intelligent packaging on the market are time-temperature indicators, freshness indicators, and leakage indicators. The indicators are classified into three groups based on their function: critical temperature indicators (CTI), critical temperature/time integrators (CTTI), and time-temperature integrators or indicators (TTI) [35].

The temperature of a food product has a significant impact on its shelf life. Deviations in the temperature profile can lead to the growth or survival of microbes, resulting in product deterioration. The exposure of a product to an incorrect temperature for a period of time sufficient to create unfavorable changes in the product’s safety or quality is shown by CTI. The most prevalent indicators in intelligent packaging are TTI. There are two sorts of such indicators: temperature indicators and time temperature indicators [35]. TTI depicts variations in ambient temperature that happened throughout the distribution and storage of food. Physical, chemical, enzymatic, or microbiological changes occur as a result of temperature changes [38]. They are utilized to keep track of a product’s present temperature and its surroundings, as well as any deviations from the ideal temperature during distribution and the simultaneous aggregate of its intensity and time of occurrence. The Monitor Mark uses a color ring or belt moving on a white background to indicate a change in the management of a product. Physical diffusion of a solution with a chemically altered color causes this. The temperature ranges for this indicator’s application vary based on the type of indicator; however, the threshold limits over which the diffusion occurs are between −17 °C and 48 °C. Freshness indicators monitor the quality of food products while they are being stored and transported. Unfavorable conditions or a lack of durability might cause a loss of freshness. Fresh-Check is based on temperature-dependent polymerization reactions that result in changes in the look of a label. This indication is shaped like a small circle surrounded by a reference color circle.

#### 3.2.3. Sensors

A sensor is a device that detects, locates, or quantifies energy or matter by sending out a signal for the detection or measurement of a physical or chemical property [40]. The majority of sensors are made up of two parts: a sensor and a receptor. Certain chemical or physical analytes can be detected by measuring their existence, activity, composition, or concentration. The receptor also converts physical or chemical information into an energy form that can be detected by the second unit—the transducer [41]. In addition, the transducer is utilized to convert the measured signal into an analytic signal that may be employed. An electrical, chemical, optical, or thermal signal could be used [42]. There are various types of sensors that monitor various characteristics, such as gas sensors, chemical sensors, and biosensors. Liu et al. [11] prepared κ-Carrageenan-mulberry polyphenol extract (MPE) active packaging films. MPE enhanced the water vapor and UV–vis light barrier ability of the κ-carrageenan film. It showed antioxidant, pH-sensitive properties, and could monitor the freshness of milk.

### 3.3. Edible Films

Edible films are packaging materials that are made of edible ingredients. Nowadays, using edible films has become an increasing demand due to their environment friendly nature, safety of consumption, and ease of use [43]. Edible films have the ability to increase food quality, freshness, and shelf-life. The edible films form a semipermeable barrier around the packaged food product, increasing its barrier properties by reducing the exchange of moisture, lipid, gas, and volatiles [44,45]. The properties of natural edible films can be enhanced by the addition of aromatic substances, flavor enhancing agents, nutritional substances, antioxidant agents, or antimicrobial agents [44]. The antimicrobial agents have strong antimicrobial potency towards foodborne bacteria resulting in reduced microbial growth of the packed food product. The antioxidant agents result in strong DPPH radical scavenging ability resulting in low lipid oxidation of the packaged food product. The reduced lipid oxidation and microbial growth results in reduced food spoilage, which ultimately leads to prolonged shelf-life of food products and increases the ability of food preservation [46,47,48]. Edible films can also be used as delivery systems for the controlled release of bioactive components, nutrients, pharmaceuticals, and food ingredients. High solubility is a very important factor when designing edible films since they have to dissolve fast [49].

The most commonly used seaweeds as edible films are Carrageenan, Sodium alginate, and Agar. This seaweed has properties such as flexibility and transparency that will aid in edible film development [48]. Many researchers have developed novel edible films with seaweeds while incorporating other biopolymers, bioactive agents with suitable properties for packaging, and increased bioactivity, as shown in Table 3. Paula et al. [50] developed a pure seaweed edible film with k-Carrageenan, i-Carrageenan, and Alginate with an increased barrier, optical and mechanical properties. Antioxidant properties were observed in a semi-refined Carrageenan and Ulvan edible film. High hydroxyl radical scavenging activity was observed in the Ulvan polysaccharide-based film while high metal ion chelating activity was observed in the combined film [52]. Agar-based edible films have been developed with the incorporation of biopolymers such as Starch, Maltodextrin [49] and Maltodextrin, beeswax, shortening, liquid paraffin [54] with improved film forming ability, mechanical properties, and hydrophobicity. Further, biopolymers such as Chitosan have been utilized with the combination of seaweeds to form edible films. Albertos et al. [4] developed an edible film with *Himanthalia elongata*, *Palmaria palmata*, and Chitosan, which increased the self-life of fish burgers during storage by reducing microbial growth and lipid oxidation. Gomaa et al. [55] also formed a chitosan based edible film with the seaweeds Alginate and Fucoidan that showed increased barrier properties and exhibited excellent antioxidant properties. Bioactive components such as tea polyphenols [43], essential oils [46], and Capsaicin [48] have been incorporated to form an Alginate based edible film with improved antioxidant and antimicrobial properties. Further, the incorporation of *Lactococcus lactis* into Sodium alginate, Sodium carboxymethylcellulose, and Collagen edible film increased the antimicrobial properties of the film [51]. Ferulic acid incorporated into Sodium alginate edible films showed increased antioxidant activity. However, it did not show any antimicrobial activity [47]. In addition to bioactive components, some non-toxic nanoparticles are also used to form edible films. Agar and nano-bacterial cellulose edible film has improved mechanical, barrier, thermal properties, and crystallinity [53]. As previously stated, seaweed-based edible films have been produced for the market in recent years.

### 3.4. Coatings

A coating is a relatively thin layered material that is directly formed on the surface of a food product for its protection and improvement in shelf-life [56]. Food coatings have various functions, such as modifying the functional attributes of foods, acting as a barrier between the environment and food products, and controlling the moisture on the food’s surface. Polyalcohols, modified starches, mono and disaccharides, silicates, and other anti-wetting coatings are primarily used for coating solid food products. In addition, polysaccharides and/or proteins are generally used for the development of edible coatings [57].

Seaweed polysaccharides such as Alginate and Carrageenan have been widely used in the development of edible coatings as presented in Table 4. The functionalities of the seaweed-based coating can be enhanced by incorporating them with phenolics, essential oils, nanomaterials, and other potential substances [45]. In a study, Nair et al. [58] reported that the alginate-based edible coating incorporated with 1% polyphenolic rich pomegranate peel extract successfully inhibited the growth of *Colletotrichum gloeosporioides* in capsicum stored at 10 °C. Further, the color, firmness, weight, total chlorophyll, and ascorbic content of capsicum were retained after coating. Moreover, the shelf-life of coated capsicum determined on the basis of sensory quality attributes was found to be 20 days, which was higher than the control samples (12 days of shelf life). In another research, Zhou et al. [59] used kappa-carrageenan (KC), konjac glucomannan (KGM), and camellia oil (CO) to develop a coating material (KGM/KC-CO) for improving the shelf life of chicken meat. The microbial counts, total volatile nitrogen, thiobarbituric acid reactive substance, pH, and weight loss were reduced significantly in the KGM/KC-CO coated samples. Further, the incorporation of 3.5% CO into the KGM/KC-based coating improved the shelf life of chicken meat by up to 10 days when stored at 4 °C.

**Figure 3:**
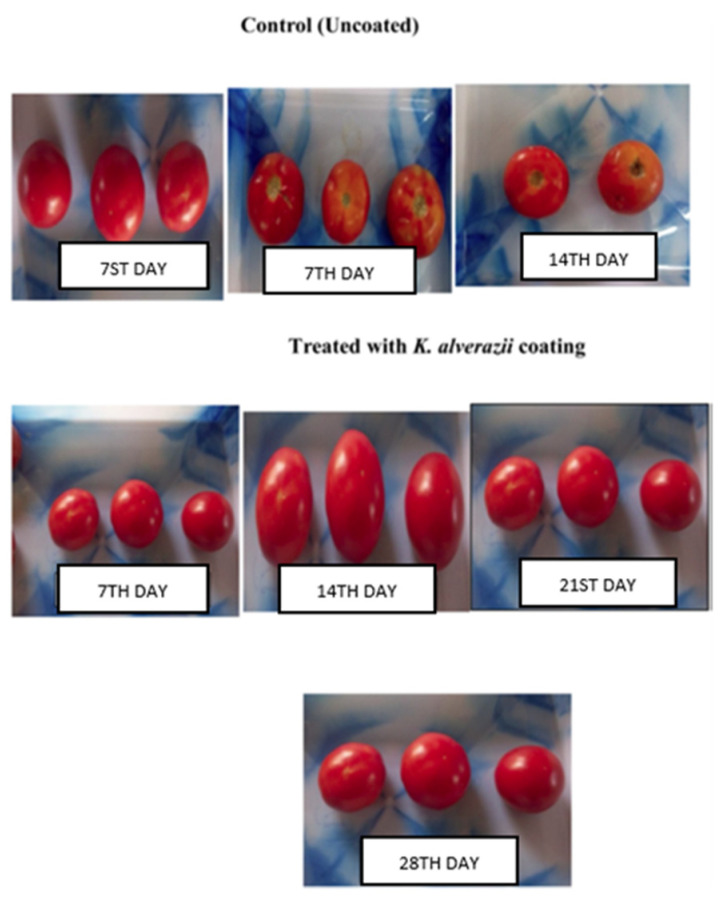
Texture of uncoated and *Kappaphycus alvarezii* (red algae) coated tomatoes during storage [61].

**Figure 4:**
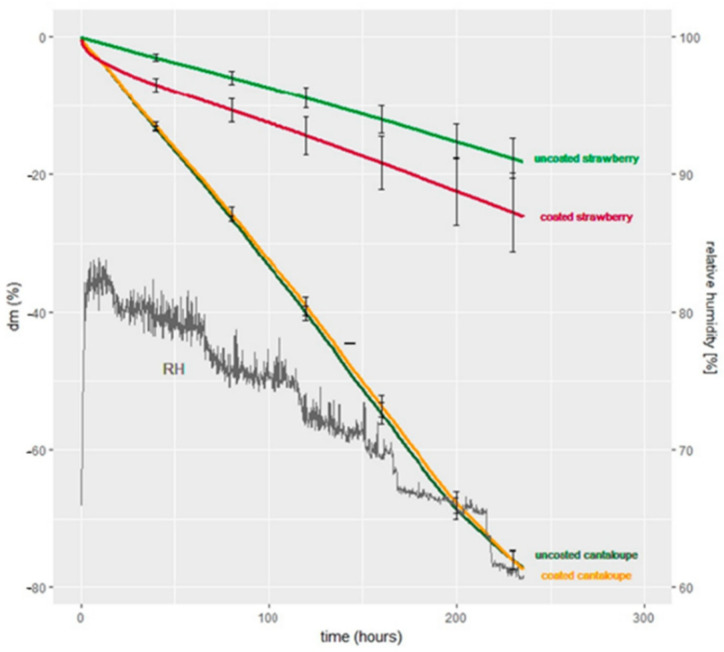
The relationship between the time and mass loss (%) of coated and uncoated strawberries and fresh-cut cantaloupes at 10 °C [65].

In recent years, crude seaweed extracts have also been used for developing edible coating materials. In a study, Banu et al. [61] prepared seaweed extract from *Sargassum tenerrimum* (brown algae) and *Kappaphycus alvarezii* (red algae) for coating of tomatoes. The phytonutrient analysis confirmed the presence of tannins, saponins, steroids, and flavonoids in both seaweeds. The antifungal and antibacterial activity of *K. alvarezii* exhibited a higher inhibition zone compared to *S. tenerrimum* against common pathogens. Tomatoes coated with 3% *K. alvarezii* extract had better quality characteristics than the uncoated and *S. tenerrimum* coated tomatoes during the 30 days of storage as shown in Figure 3. Compared to the uncoated samples, the lowest reduction in juice content, ascorbic acid content, and total acidity was recorded in 3% *K. alvarezii* coated samples. Further, the 3% *K. alvarezii* extract coated tomatoes had the lowest weight loss and a higher level of total soluble solid content than the uncoated and other coated samples with different concentrations of seaweed extract. In another study, red seaweed (*Gracilaria gracilis*) extract (RSE) was used to enrich the microalgal exopolysaccharides (EPS) based edible coatings for the preservation of shrimp under refrigerated storage (4 ± 1 °C). The EPS + RSE coating led to a significant reduction in thiobarbituric acid reactive substances, trimethylamine content, and total volatile base values during the 8 days of storage. Enrichment of EPS with 1 and 1.5% of RSE was most effective in inhibiting the growth of psychrotrophic bacteria. The coated samples had better oxidative stability and lower polyphenol oxidase activity at the end of the storage period. Further, improvement in the color and hardness of the shrimp was observed while initial sensory characteristics were retained after coating with EPS + RSE [60]. Parreidt et al. [65] developed a sodium alginate-based edible coating for fresh-cut cantaloupe and strawberries. Here, the coating of strawberry promoted the water loss of strawberry while it reduced the water loss of cantaloupe pieces as depicted in Figure 4. The reduced water loss of the sample is dependent on the water vapor transfer permeability.

## 4. Effects of Seaweeds on Food Contact Materials

### 4.1. Mechanical Properties

Mechanical parameters such as tensile strength, elastic modulus, and elongation at break determine the quality of polymer films suitable for food packaging. These measurements show the film’s capacity to tolerate various stresses that occur during the preparation, handling, and storage of packaged food while maintaining its integrity. The mechanical properties of composite films produced through the blending process are mostly determined by the linkages and solubility of the compounds and also by intermolecular interactions between polymer chains. Moreover, the presence of integrated compounds can produce structural changes in the film matrix, resulting in a less dense structure that could permit increased interaction between components, rather than only hydrogen interactions with water molecules. The impact of seaweed polysaccharide inclusion on mechanical quality has also been investigated in several studies. The integration of seaweed polysaccharides into the corresponding polymer resulted in an increase in tensile strength in more than half of the analyzed experiments. Alginate and starch were utilized to form food packaging films with excellent mechanical properties [66]. Agar/Starch-based films, such as Alginate-based films, have excellent mechanical characteristics [67].

Rhim [68] has developed a multilayer film composed of Poly lactic acid (PLA) and agar/κ-Carrageenan/Clay nanocomposite films and observed the effect of lamination of PLA layers. They observed a significant increase in the tensile strength of the Agar/κ-Carrageenan/Clay nanocomposite films (67.8 ± 2.1 MPa) as compared to the PLA films (43.3 ± 3.6 MPa). Huq et al. [69] have formed a Nanocrystalline cellulose reinforced alginate-based nanocomposite film by solution casting. The study suggested that the nanocomposite reinforced with 5 wt% nanocrystalline cellulose content exhibits 37% greater tensile strength as compared to the control. However, Goonoo et al. [70] have also developed a blend of polysaccharide content containing fucoidan and polyhydroxybutyrate-co-valerate. The tensile strength was observed at 27 °C, 60% relative humidity, and a crosshead speed of 10 mm/min. For the Fucoidan/Polyhydroxybutyrate-co-valerate blend, a decrease in tensile modulus and therefore increased flexibility was observed.

### 4.2. Physical Properties

Morphological changes following the addition of components such as seaweed polysaccharides are difficult to speculate since they are highly dependent on the interaction between the polymer and the seaweed. They are, though, worth investigating because the integration of different substances could result in gaps and other changes that could affect engineering properties in the future. The physical properties of Alginates have been demonstrated to be dependent on the monomer sequence. The preferential binding of multivalent cations, which is the basis for gel formation, and the fact that the sol/gel transition of Alginates is unaffected by temperature are the most significant physical properties of Alginate [71]. Jumaidin et al. [72] formed a sugar palm starch, and agar film with different concentrations of Agar studied the surface morphology with scanning electron microscopy (SEM). The study suggested, after adding Agar to the mix, the SEM image exhibited a uniform and smooth surface with no clusters and no phases of sugar palm starch, indicating that the two film components are miscible and interact effectively. However, when the Agar concentration increased, breakage structures appeared on the surface, which could be due to polymer-polymer bonding and a larger filler content in the film matrix.

SEM examination of Polyhydroxy butyrate film samples was utilized by Lopes et al. [73] to investigate the morphological changes caused by esterified alginate added to the Polyhydroxy butyrate blend. After the polysaccharide was added, the morphological structure of the Polyhydroxy butyrate blend changed, revealing a dense, rough, and open texture on the surface. Eghbalifam et al. [74] produced a Poly vinyl alcohol/Sodium alginate film and studied the surface morphology of the films. The blend film had a homogeneous texture, indicating excellent miscibility and no phase separation, according to the SEM image analysis.

### 4.3. Chemical Properties

Chemical characteristics such as hydrophilicity or hydrophobicity, as well as the composition of a film with functional groups in FCM, all play a critical part in determining the safety and efficiency of FCMs [3]. In a study, Kadam et al. [75] used the sessile drop technique to measure the static contact angle of Gelatin film and Sodium caseinate films incorporated with brown seaweed *Ascophyllum nodosum* (*A. nodosum*) extract. The addition of seaweed extract resulted in the reduction of the contact angle. For both the films, the hydrophilicity increased with the incorporation of a higher amount of seaweed extract. Further, the polar component of the surface free energy increased significantly with the addition of seaweed extract. The presence of a high number of hydroxyl groups in the seaweed extract resulted in an increase in the polar component of surface free energy, which contributed to the increase in the films’ hydrophilicity. In another research, the Carrageenan-based composite films’ hydrophobicity was greatly influenced by the incorporation of grapefruit seed extract (GSE). The control carrageenan film’s water contact angle (WCA) was higher than the Carrageenan/GSE composite film. With the increase in GSE concentration, the WCA of the composite films was reduced significantly, which was due to the GSE’s hydrophilic nature [76].

**Figure 5:**
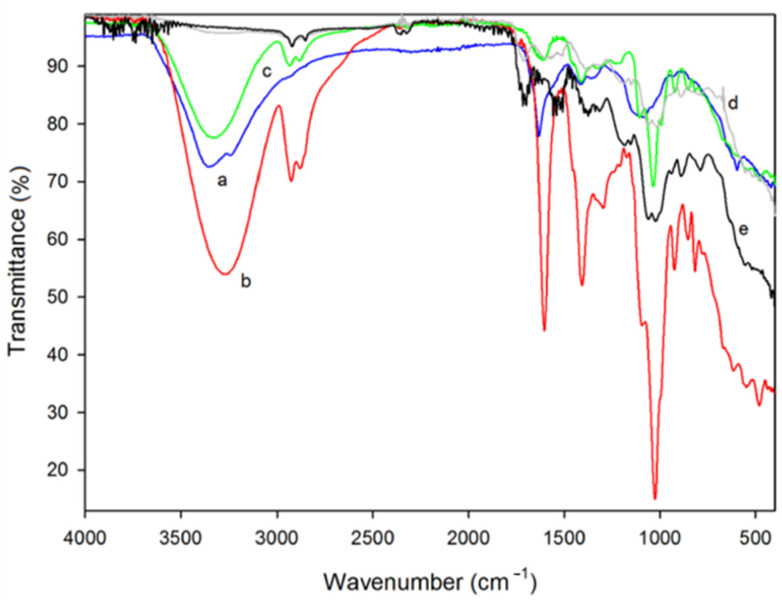
Fourier transform infrared spectroscopy attenuated total reflection (FTIR-ATR) spectrum of the (a) *C. tomentosum* seaweed extract (blue) and the different tested films: (b) 1% alginate (red), (c) 1% alginate with 0.5% of *C. tomentosum* seaweed extract (green), (d) 1% chitosan (grey), and (e) 1% chitosan with 0.5% of *C. tomentosum* seaweed extract (black) [78].

The Fourier transform infrared spectroscopy (FTIR) technique has been extensively utilized to characterize various polymers and their composites. It has been observed that the interaction of chemical groups at the molecular level leads to shifting of the absorption band in the infrared spectrum, which indicates that the polymer is miscible [77]. In a study, Liu et al. [11] used FTIR spectroscopy to examine intermolecular interactions between mulberry polyphenolic extract (MPE) and κ-carrageenan matrix. Compared to the κ-carrageenan film, broader bands at about 3340 cm^−1^ were observed in κ-carrageenan-MPE films, indicating the intermolecular hydrogen bonding between hydroxyl groups of MPE and κ-carrageenan. Further, the incorporation of MPE led to the shifting of the band at 1222 cm^−1^ to 1220–1221 cm^−1^, suggesting intermolecular interactions between –SO_3_^−^ groups of κ-carrageenan matrix and MPE. In another study, FTIR analysis of Chitosan-based edible films incorporated *Himanthalia elongate* seaweed (FH) and *Palmaria palmata* seaweed (FP), and their extracts (FHE and FPE) showed characteristic absorption bands of only chitosan. However, the FTIR spectra did not clearly indicate the existence of seaweed extracts in the films, which might be due to the similarity between the functional groups of seaweeds and chitosan [4]. As per the FTIR bands in Figure 5, the *C. tomentosum* seaweed extract and sodium alginate showed absorption bands at 1100–930 cm^−1^ representing C–C, C–O pyrenoid ring stretching, and C–O–C glycosidic bond stretching. The interaction of sodium alginate, chitosan, and *C. tomentosum* seaweed extract is observed in bands’ areas [78].

### 4.4. Thermal Properties

Thermal analysis techniques are helpful in determining the changes in material properties through the heating process [79,80]. The differential scanning calorimetry (DSC) and thermogravimetry analysis (TGA) methods are widely utilized to investigate the thermal profiles of polymers and their composites [81]. DSC is used to measure the difference in the quantity of heat needed to raise the temperature of the material and the reference as a function of temperature and time [82]. Thermal properties such as glass transition temperature ($T_{g}$), melting temperature ($T_{m}$), and crystallization temperature ($T_{c}$) can be determined from a DSC measurement [81]. DSC analysis of chitosan-based edible films incorporated with seaweeds (FH, FP) and seaweed extracts (FHE, FPE) had higher endothermic peak specific energy values (170.9–197.1 J/g) compared to the chitosan-only films (<100 J/g). Therefore, the water absorption capacity of the films as well as the interactions between the polymer and water are improved with the incorporation of seaweeds. Further, the $T_{g}$ of the edible films are not affected by the seaweed incorporation. However, the addition of seaweed did not negatively influence the $T_{g}$ of Chitosan films [4]. In another research, the DSC analysis of Gelatin films incorporated with *A. nodosum* extract showed two characteristics transition between 53.6 and 58.9 °C and 124.3 and 132.3 °C. The addition of seaweed extract did not significantly influence the first transition. However, for the second transition, a substantial increase in the transition temperature was noticed after seaweed inclusion. The stability of the film network might have been improved because of the non-disulfide covalent bond after seaweed addition, resulting in an increase in the transition temperature of the films. Moreover, the $T_{g}$ of the Sodium caseinate films reduced substantially after the addition of *A. nodosum* extract, which might be due to the plasticizing behavior of seaweed extract’s low-molecular weight components [75].

**Figure 6:**
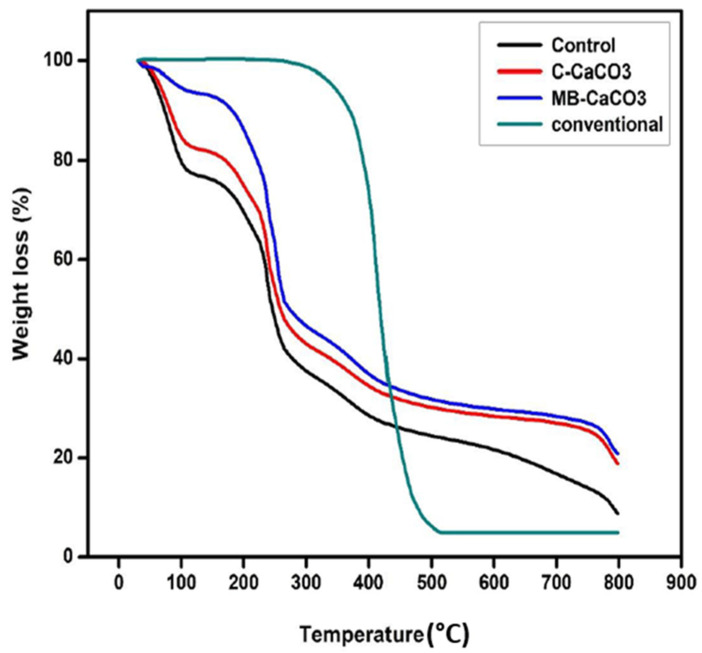
Thermogravimetric analysis (TGA) curves of control, commercial calcium carbonate (C–CaCO_3_)-based red seaweed (*Kappaphycus alvarezii*), microbially induced precipitated calcium carbonate (MB–CaCO_3_)-based red seaweed (*Kappaphycus alvarezii*), and conventional films [83].

In TGA, the thermal stability of a material can be evaluated by recording the weight change with respect to temperature under constant heating rate or time under isothermal conditions in a controlled environment. The mass gain or loss of the material due to decomposition, oxidation, or loss of volatiles can be measured using TGA [81,82]. In a study, Liu et al. [11] used TGA to determine the thermal stability of κ-Carrageenan film and κ-Carrageenan–MPE (mulberry polyphenolic extract) film. The κ-carrageenan film incorporated with 2 and 4 wt. % of MPE showed higher thermal stability than pure κ-carrageenan film. The weight loss of the film at 60–120 °C was due to the evaporation of chemisorbed water. The second stage of weight loss was observed between 121 and 230 °C, which was associated with the loss of MPE and glycerol. The final stage of weight loss was between 231 and 800 °C, which was attributed to κ-carrageenan decomposition. In another research, TGA of chitosan-based edible films containing seaweeds (FH, FP, FHE, and FPE) exhibited the first stage of weight loss between 50 and 125 °C, which was as a result of evaporation of acetic acid and water. Further, the degradation of seaweed and chitosan contributed to the second stage of weight loss of edible films. A degradation step with a temperature greater than 800 °C was also observed in the films containing FH, FP, and FHE seaweeds which confirmed that the thermal properties of the edible films were improved by the incorporation of seaweeds [4]. The thermal stability of the seaweed based materials is usually lower than the commercially available polymers such as poly lactic acid, as depicted in Figure 6 [83].

### 4.5. Antimicrobial Properties

Antimicrobial properties are an important property that can be used in FCMs to improve food safety. Antimicrobial properties allow the reduced/inhibited growth of microorganisms, thus preventing food spoilage, reducing food waste, and increasing the shelf-life of the food product [3].

Seaweeds consist of chemical compounds such as phenols, fatty acids, carbohydrates, and proteins that are responsible for antimicrobial activity. Some of the isolated compounds from seaweed that exhibit antimicrobial activity are essential oil extracted from *Undaria Pinnatifida* exhibiting antimicrobial activity against *S. aureus* and *S. Typhimurium* [84], Fucoidan exhibited antimicrobial activity against *E. coli*, *S. aureus*, *P. aeruginosa*, *A. hydrophila* [85,86,87], and *Sargassum Myriocystum* exhibited antimicrobial activity against *Vibrio parahemolyticus*, *Vibrio* and *A. hydrophilla* [88]. *Kappaphycus alvarezii* red seaweed extract when incorporated into poly vinyl alcohol retained its antimicrobial activity against *S. aureus* and *B. cereus* [89]. Research is limited on these crude seaweed extracts as antimicrobial agents in food packaging.

However, some studies have demonstrated that the seaweed polysaccharide itself doesn’t have any significant antimicrobial activity. The Sodium alginate- Ferulic acid film did not show antimicrobial activity against *Klebsiella pneumonia* and *Salmonella enterica* [47].

Essential oils, plant extracts, or nanoparticles have been incorporated into seaweeds as antimicrobial agents to increase their antimicrobial activity. The Carrageenan–Starch film incorporated with Carvacrol showed complete inhibition of *S. aureus*, while the film with Carvacrol showed no antimicrobial activity [90]. Further, the Carrageenan-based packaging material was prepared by the addition of olive leaf extract, which reduced the bacterial growth of psychrophiles up to five-fold during lamb meat storage [91]. Bhutiya et al. [92] created an active food packaging material by incorporating zinc oxide nanorods into cellulose isolated from the green seaweed *Ulva fasciata*. This packaging material showed antimicrobial activity against both gram-positive (*S. aureus*, *B. ceresus*, *S. thermophilis*) and gram-negative (*E. coli*, *P. aeruginous*) microorganisms.

### 4.6. Antioxidant Properties

The antioxidant properties of food contact materials are critical for reducing lipid oxidation and protein degradation in packaged foods. Lipid oxidation leads to reduced shelf-life, quality, nutritional value and negatively affects the appearance of the food products. Lipid oxidation is a major cause of food spoilage that results in a large amount of food waste. Thus, during active packaging, the antioxidant properties of the materials are increased by the incorporation of antioxidants into the films/coatings [3,11]. These incorporated antioxidant agents can minimize the problems associated with lipid oxidation, and they also progressively migrate to the surface of the food product during storage [29].

Most of the seaweeds used as FCMs have some antioxidant properties. For instance, Kanatt et al. [89] studied the bioactive properties of *Kappaphycus alvarezii*, red seaweed, and this film exhibited good antioxidant activity when measured by the DPPH (2,2-diphenyl-1-picrylhydrazyl) assay. The addition of *Fucus vesiculosus* seaweed extract to a Whey protein film increased the antioxidant capacity while inhibiting lipid oxidation in the chicken breast up to 25 days of storage [29].

However, these antioxidant properties of the materials are enhanced by the addition of essential oils, and plant extracts that can reduce the lipid oxidation of the food product. Tea polyphenols were added to the Gelatin–Sodium alginate film that increased the antioxidant activity of the film [43]. The addition of Ferulic acid to a Sodium alginate edible film increased the antioxidant capacity of the film by a few folds [47]. In addition, studies were conducted on Carrageenan films with the incorporation of different levels of olive leaf extract. The increasing level of olive leaves extract increases the antioxidant properties of the films [93]. Further, studies have been conducted on the incorporation of antioxidant agents mulberry polyphenol extract [11] and pomegranate flesh/peel extract [77] in the κ-Carrageenan films. The addition of these antioxidant agents also increased the antioxidant capability of the films, while the antioxidant activity increased with increasing concentrations. These incorporated antioxidant agents have the ability to be gradually released onto the food surface, reducing lipid oxidation, food spoilage and in return, increasing food quality and shelf-life.

## 5. Legal Aspects of Using Seaweed as Food Contact Material

FCMs and articles (e.g., food packaging) are a relevant pathway for the exposure of food contact chemicals (FCC) to consumers. The FCC includes both known and uncharacterized chemical substances, which might pose a significant risk to human health [94]. With regards to FCMs, the European Union regulation EC/1935/2004 (Article 3(1)(a)) requires that “materials do not release their constituents into food at levels harmful to human health” [95]. A total of 8030 substances that can be utilized in various food contact articles are listed in the EU and EU Member State regulations. Additionally, around 10,787 substances (roughly half are FCC) can be utilized directly or indirectly as food additives in the USA [94].

Seaweeds are increasingly being utilized as FCMs due to their numerous benefits. However, they can accumulate several hazardous compounds and toxic metals, including mercury, lead, arsenic, etc. [3]. Cadmium is naturally accumulated in seaweed. Thus, food supplements containing dried seaweed, or its derivatives may have a higher amount of cadmium than other food supplements. According to Commission Regulation (EC) No 629/2008 of 2 July 2008, amending Regulation (EC) No 1881/2006 setting maximum levels for certain contaminants in foodstuffs, a maximum amount of 3 mg cadmium/kg dry weight of seaweed is allowed [96]. Maximum arsenic content of 40 mg/kg and 10 mg/kg (moisture content of seaweed 12%) is allowed in complementary seaweed feed/feed meals according to the Commission Regulation (EU) No 1275/2013 of 6 December 2013 amending Annex I to Directive 2002/32/EC of the European Parliament (EU, 2013). In other parts of the world, such as South America, the USA, and Asian countries, there is a lack of legislation regulating the concentration of toxic substances in seaweed or seaweed-derived products [3].

## 6. Conclusions and Future Perspective

There is an increased awareness about the use of sustainable FCMs for environmental and health benefits. The utilization of seaweed in developing FCMs has sparked considerable scientific interest due to several beneficial characteristics of seaweeds such as biodegradability, non-toxicity, transparency, barrier properties, film-forming ability, and antioxidant/antimicrobial properties. The mechanical, chemical, physical, thermal, antioxidant, and antimicrobial properties of seaweed based FCMs have been substantially improved by using raw seaweed extract or its polysaccharide in combination with other biopolymers and additives such as nanoparticles and active agents. Although the utilization of seaweed as an FCM is gaining traction in the food industries, more research is required to determine how these materials can be further optimized and improved to enhance shelf-life and maintain the quality of the food products under different transportation and storage conditions. Furthermore, the development of appropriate regulatory policies and standards would help in the commercialization of seaweed based FCMs. However, the major drawback towards seaweed commercialization is the high cost of production when compared to the packaging materials currently used in food industries. The production cost can be reduced by increasing the farming and cultivation of seaweeds along with developing associated processing technologies.

## Figures and Tables

**Table 1:** Seaweed polysaccharides commonly used as food contact materials.

Seaweed	Polysaccharide	Structure	Reference
Brown seaweeds	Alginate	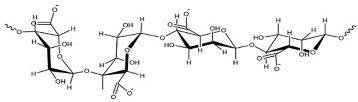	[3]
Red seaweeds	Agar	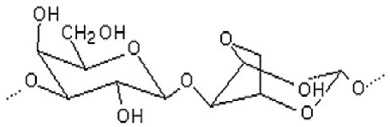	[3]
	Carrageenan	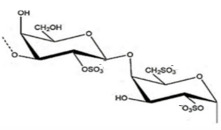	[3]
Green seaweeds	Ulvan	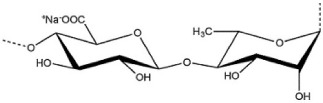	[3]

**Table 2:** Recent application of seaweed polysaccharides in active and intelligent packaging.

	Packaging Matrix	Properties of the Packaging Material	Reference
Active packaging			
Sodium alginate	Lemongrass oil	Controlled release of lemon grass oil due to microencapsulation Films were able to inhibit growth of *E. coli* and *L. monocytogenes*	[31]
Furcellaran	Gelatin hydrolysate/Rosemary extract	Increased thickness, water content, and tensile strength Colour changes in different pH were observed High antioxidant activity.	[10]
Sodium alginate	Carboxymethyl cellulose, CaCl_2_, Pyrogallic acid	Higher elongation at break Increased moisture content, water vapor permeability, and oxygen permeability Effective against *E. coli* and *S. aureus*	[30]
Gelidium sesquipedale	Polyvinyl alcohol, Nanocellulose	Enhanced properties for the incorporation and release of bioactive extracts.	[32]
*Fucus vesiculosus*	Whey protein	Strengthened the thickness, tensile strength, and elastic modulus Inhibit lipid oxidation of packaged poultry meat, at least for 25 days of storage	[29]
Smart and intelligent packaging			
κ-Carrageenan	Mulberry polyphenol extract	Increased water vapor and UV–vis light barrier ability Improved antioxidant and pH-sensitive properties Could be used to monitor the freshness of milk.	[11]
Carrageenan	Gelatin, Shikonin, and Propolis	Improved UV barrier without reducing film transparency Excellent pH-responsive color change over a wide pH range of 2–12 Intelligent film was effective in monitoring the freshness of packaged milk	[33]
κ-Carrageenan, agar	Nano-TiO_2_, nano-ZnO, and *Clitoria ternatea Linn* anthocyanin	Increased UV–vis light barrier, pH sensitivity, and physical properties. In the buffer solution (pH 2.0–12.0), ammonia vapour (80 M), and pork spoilage studies, films showed visual color changes.	[12]

**Table 3:** Instances of seaweed polysaccharides in edible films.

	Packaging Matrix	Properties of the Packaging Material	Reference
k-carrageenan, i-carrageenan, and Alginate	Glycerol	k-carrageenan improved moisture barrier and tensile properties Alginate favoured film uniformity and transparency I-carrageenan effect the opacity of film	[50]
Sodium alginate	Gelatin- tea polyphenols	Increased tensile strength, contact angle, and cross-linking Decreased elongation at break and water vapor permeability Increased antioxidant and physical properties with increased tea polyphenol levels.	[43]
Sodium alginate	Sodium carboxymethylcellulose, collagen, and *Lactococcus lactis*	Reduced gloss and transparency when incorporation of *Lactococcus lactis* Inhibition of *S. aureus* growth for 7 days	[51]
Sodium alginate	Essential oils of *R. officinalis* L., *A. herba alba Asso*, *O. basilicum* L. and *M. pulegium* L.	Strong antibacterial activity against six pathogenic bacteria with inhibition zone between 18.5 and 38.67 mm Antioxidant capacity of the films ranged from 4.57% to 23.09% Improved thermal and barrier properties Decreased tensile strength Film is biodegradable in the soil.	[46]
Alginate	Pullulan and Capsaicin	Decreased the transmittance, elongation at break, and moisture content with increasing levels of Capsaicin Increased tensile strength, water vapour permeability, and surface contact angle Exhibit good antibacterial activity against *E. coli* and *S. aureus*	[48]
Sodium alginate	Ferulic acid	Increased antioxidant activity with increased concentration Ferulic acid No zone of inhibition against *Klebsiella pneumonia* and *Salmonella enterica*.	[47]
Semi refined carrageenan and ulvan	Glycerol	A strong hydroxyl radical scavenging activity in ulvan polysaccharide-based film High metal ion chelating activity in Semi refined carrageenan and ulvan films Low molecular weight films had better antioxidant activity High molecular weight films had good mechanical properties	[52]
Agar	Starch, and Maltodextrin	Improved film forming ability and hydrophobicity Interaction between starch and agar led to poor water solubility Maltodextrin results in highly miscible and plasticized starch-agar films Increased solubility with higher Maltodextrin concentration	[49]
Agar	Nano-bacterial cellulose	Improved crystallinity and thermal stability Bacterial cellulose decreased moisture content, water solubility and water vapour permeability Increased tensile strength from 22.10 to 44.51 MPa	[53]
Agar	Maltodextrin, beeswax, shortening, and liquid paraffin	Incorporation of hydrophobic agents significantly improved the hydrophobicity of films The tensile strength, elongation at break and Young’s Modulus of the films increased when hydrophobic agents are incorporated	[54]

**Table 4:** Instances of seaweed polysaccharides in coatings.

	Packaging Matrix	Properties of the Packaging Material	Reference
Alginate	Pomegranate peel extract	Inhibition against *Colletotrichum gloeosporioides* and improvement in shelf life of capsicum.	[58]
Kappa-carrageenan	Konjac glucomannan and camellia oil	Reduction in microbial count and improvement in shelf life of chicken meat by up to 10 days when stored at 4 °C.	[59]
*Gracilaria gracilis* extract	Microalgal exopolysaccharides	Inhibition against psychotropic bacteria and retention of sensory quality during 8 days of refrigerated storage.	[60]
*Sargassum tenerrimum* and *Kappaphycus alvarezii* extract	-	*K. alvarezii* had better inhibition against common pathogens compared to *S. tenerrimum* coated tomatoes. Best retention of quality in 3% *K. alvarezii* extract coated samples.	[61]
κ-carrageenan	Chitosan	Reduction in weight loss and positive effect on reducing disease infection of dragon fruit stored at 10 °C, 90–95% RH. Retention of freshness and bract chlorophyll content for 30 days.	[62]
Sodium alginate	Oregano essential oil (1.5–2.5% *w*/*w*), mandarin fibre (0.5% *w*/*w*)	Effective decontamination of pathogens such as *Staphylococcus aureus* Extended shelf life of low-fat cut cheese	[63]
Sodium alginate	Pomegranate peel extract, Chitosan	Improved the visual attributes of guava Efficiently retained the nutritional parameters Delayed senescence	[45]
Carrageenan	Chitosan	Decreases in water loss, weight loss, and respiratory rate in coated fruits Preserve the longan by showing minimal quality changes and quantity losses	[64]

## Data Availability

Data sharing not applicable.
